# Assessing HCH isomer uptake in *Alnus glutinosa*: implications for phytoremediation and microbial response

**DOI:** 10.1038/s41598-024-54235-1

**Published:** 2024-02-20

**Authors:** Aday Amirbekov, Stanislava Vrchovecka, Jakub Riha, Ivan Petrik, David Friedecky, Ondrej Novak, Miroslav Cernik, Pavel Hrabak, Alena Sevcu

**Affiliations:** 1 Institute for Nanomaterials, Advanced Technologies and Innovation, Technical University of Liberec, 460 01 Liberec, Czech Republic; 2 Faculty of Mechatronics, Informatics and Interdisciplinary Studies, Technical University of Liberec, 461 17 Liberec, Czech Republic; 3 Laboratory of Growth Regulators, Institute of Experimental Botany, Czech Academy of Sciences and Faculty of Science, Palacký University Olomouc, 78371 Olomouc, Czech Republic; 4 Laboratory for Inherited Metabolic Disorders, Department of Clinical Biochemistry, University Hospital Olomouc and Faculty of Medicine and Dentistry, Palacký University Olomouc, 775 20 Olomouc, Czech Republic; 5 Faculty of Science, Humanities and Education, Technical University of Liberec, 460 01 Liberec, Czech Republic

**Keywords:** Environmental biotechnology, Plant biotechnology, Chemical biology

## Abstract

Although the pesticide hexachlorocyclohexane (HCH) and its isomers have long been banned, their presence in the environment is still reported worldwide. In this study, we investigated the bioaccumulation potential of α, β, and δ hexachlorocyclohexane (HCH) isomers in black alder saplings (Alnus glutinosa) to assess their environmental impact. Each isomer, at a concentration of 50 mg/kg, was individually mixed with soil, and triplicate setups, including a control without HCH, were monitored for three months with access to water. Gas chromatography–mass spectrometry revealed the highest concentrations of HCH isomers in roots, decreasing towards branches and leaves, with δ-HCH exhibiting the highest uptake (roots—14.7 µg/g, trunk—7.2 µg/g, branches—1.53 µg/g, leaves—1.88 µg/g). Interestingly, α-HCH was detected in high concentrations in β-HCH polluted soil. Phytohormone analysis indicated altered cytokinin, jasmonate, abscisate, and gibberellin levels in A. glutinosa in response to HCH contamination. In addition, amplicon 16S rRNA sequencing was used to study the rhizosphere and soil microbial community. While rhizosphere microbial populations were generally similar in all HCH isomer samples, Pseudomonas spp. decreased across all HCH-amended samples, and Tomentella dominated in β-HCH and control rhizosphere samples but was lowest in δ-HCH samples.

## Introduction

Pesticides were invented for widespread use in agriculture to control pests; however, in trying to solve one problem, several new ones were spawned. The widespread production and use of lindane (γ- hexachlorocyclohexane [HCH]), for example, has polluted soil, water and atmospheric systems worldwide^1–3^. While there are other HCH isomers, only γ-HCH was used as a commercial pesticide; nevertheless, production and purification of γ-HCH results in the formation of numerous other waste residual isomers and chlorobenzenes (ClB). It has been calculated, for example, that production of approx. 600,000 tonnes of γ-HCH generates 4.8 to 7.2 million tonnes of HCH/ClB waste, much of which is discharged close to the place of production, buried underground or kept in storehouses^4–6^. In Europe, 299 HCH polluted sites were identified within the inventory project^7^. Among these, 54 were identified as former production sites, posing elevated risks and the potential for significant impacts on both human and environment. Owing to their persistence and potential toxicity in the environment, numerous studies have examined the chemical reduction^8–11^ and biodegradation of HCH isomers^12–14^, including phytoremediation to optimise remediation technologies^15,16^.

The isomerisation rate of individual HCH isomers, which is dependent on isomer stability, will have a strong influence on the success of bioremediation at contaminated sites. As a result, isomerisation, which can take place through biotic or abiotic mechanisms^17–19^, may either promote or prevent successful bioremediation at such sites, depending on the stability of the isomers created and those converted.

While HCH isomers display low bioavailability, they tend to sorb onto organic materials in the environment, depending on local conditions. The uptake of HCH by plants, for example, will depend on several conditions, such as the isomer’s physicochemical properties, soil type, plant species and climatic factors^20^. HCH compounds and most of their metabolites are highly hydrophobic and, as such, tend to concentrate in the roots, with just a little translocated to shoots^21,22^. Overall, HCH isomers display high lipophilicity, with individual isomers having log K_ow_ partition coefficients of α-HCH = 3.8; β-HCH = 3.78; γ-HCH = 3.72; δ-HCH = 4.14 and ε-HCH = 4.19^23,24^. Consequently, uptake of HCH will be low, implying high levels of biodegradation in the plant’s rhizosphere. Indeed, HCH uptake and transformation is facilitated by plant rhizosphere and endosphere microorganisms^25–27^, which create more polar metabolites, such as ClB, that can then more easily enter the root system and translocate to other parts of the plant^28,29^.

A number of bacterial strains isolated from polluted soils (mostly *Sphingomonas*) are capable of degrading HCH isomers under aerobic conditions^30–33^. Plants release various substances into the soil, such as root exudates, mucigel and root lysates^34–36^, that provide a nutrient-rich habitat for microorganisms that enhance co-metabolic transformation of pollutants, stimulate genes encoding enzymes necessary in the degradation process and increase surfactant activity^37–39^. Theoretically, therefore, phytostimulation of rhizospheric HCH-degrading bacteria could prove effective in the remediation of HCH-contaminated soils^40^. Indeed, soil inoculation with rhizospheric microorganisms has already been shown to enhance HCH degradation^15,41,42^. The bacterial genes that encode enzymes involved in the HCH degradation process include *linA* (encoding dehydrochlorinase), *linB* (halidohydrolase), *linC* (dehydrogenase), *linD* (reductive dechlorinase) and *linE* (ring-cleavage oxygenase), all of which are involved in the complete mineralisation of γ-HCH^43,44^. The lin signalling pathway, first described in *Sphingobium japonicum* UT26, comprises an upstream (conversion of HCH to 2,5-dichlorohydroquinone (2,5-DCHQ)) and a downstream (conversion of 2,5-DCHQ into tricarboxylic acid cycle intermediates) component, and both these degradation pathways have been described for HCH compounds by Lal et al.^44^. Trantirek et al.^45^ have shown that LinA demonstrates stereoselective dehydrochlorination at a 1,2-biaxial pair of hydrogen and chlorine. This configuration is found in α-, γ-, and δ-HCH isomers. With minimal information available, the substrate range of LinA has mostly been investigated qualitatively, suggesting probable selectivity towards α-, γ-, and δ-HCH and their related PCCH (pentachlorocyclohexane) products^46^. The hydrolytic dechlorination of 1,4-TCDN (1,3,4,6-tetrachloro-1,4-cyclohexadiene) to 2,5-DDOL (2,5-dichloro-2,5-cyclohexadiene-1,4-diol) is attributed to the *linB*-encoded halidohydrolase (LinB). LinB, on the other hand, has been reported to catalyze the hydrolytic dechlorination of β- and δ-HCH, albeit at substantially lower efficiencies, and recent discoveries reveal some action against the α-isomer in LinB from strain B90A^47,48^. Furthermore, LinR induces LinD transcription in strain UT26 when appropriate substrates such 2,5-DCHQ (LinD) and CHQ (LinD) are present^49^. Interestingly, Suar et al. have discovered that α- and γ-HCH can stimulate the *linD* and *linE* genes in strain B90A, but not β- and δ-HCH^50^.

Plants have developed sophisticated hormonal mechanisms that allow them to recognise stress signals and provide the best possible response, allowing them to adapt to unfavourable conditions. Nine phytohormone categories have been identified so far, i.e. auxins (AUX), cytokinins (CK), gibberellins (GA), ethylene (ETH), brassinosteroids (BR), strigolactones (SL), abscisic acid (ABA), salicylates (SA) and jasmonates (JA), all hormones involved in the stress response. In our previous study on the effect of HCHs on the germination, early development and remediation potential of different progeny from maternal *A. glutinosa* trees^51^, we were able to demonstrate an effect of HCH on selected phytohormones. In this case, the concentrations of growth (Indole-3-acetic acid (IAA)) and stress (JA, SA and ABA) hormones were altered compared to the control group following addition of HCH, depending on the type of progeny.

As native species are already adapted to unfavourable local conditions, they could prove effective as a means of removing contaminants. As an example, black Alder (*Alnus glutinosa*) is the dominant tree species at a site in the Czech Republic highly contaminated with HCHs (50° 17′ 31.5″ N 12° 53′ 35.2″ E). The species is a widespread, short-lived tree that grows in low-lying, moist areas and, as such, has proven useful for flood control, riverbank stabilisation and river ecosystem functioning. Desai et al. were able to show that the species was effective at taking up lead (Pb) from contaminated soils^52^, while Tischer & Hübner proposed its use for degrading mineral oil hydrocarbons, phenol and polycyclic aromatic hydrocarbons (PAHs) through promotion of microbial activity via mycorrhizal nitrogen fixation^53^. While, to the best of our knowledge, no full-scale study has yet been published on HCH uptake by *A. glutinosa*, our own preliminary studies have demonstrated that presence of 12-day-old *A. glutinosa* seedlings enhances δ-HCH removal by 21–36% compared to control plots without seedlings^51^.

In this study, we aim to describe the uptake and transformation of HCH isomers by *A. glutinosa* saplings planted in freshly contaminated soil and compare the development of associated rhizosphere and soil microbial communities. In doing so, we also determine the physiological response of *A. glutinosa* to HCH isomers by measuring sapling biomass and phytohormonal activity. We hypothesise that:

i. δ-HCH isomer will demonstrate lowest bioaccumulation in *A. glutinosa* saplings (similarly to the unpublished field data for 20 year old *A. glutinosa* at HCH site Hájek, CZ).
ii. Highest HCH concentrations will be found in the root system of *A. glutinosa* saplings, with decreasing concentrations in the trunk, branches and leaves, reflecting the plant’s uptake and translocation mechanisms.
iii. The introduction of HCH isomers will lead to changes in plant physiology status and in the composition and abundance of microbial communities, with specific bacterial species responding differently to different HCH isomers, potentially indicating their ability to degrade or interact with the contaminants.

## Material and methods

### Chemicals

Standard α-HCH, β-HCH and δ-HCH isomers, along with LC grade acetone and hexane, were purchased form Merck (Merck, St. Louis, Missouri, United States), while the deuterated γ-HCH standard and ClB standards were obtained from Neochema (Neochema GmBH, Bodenheim, Germany). Anhydrous sodium sulphate was provided by Lach-Ner s.r.o. (Neratovice, Czech Republic). Deionised water was prepared using the Millipore Direct Q® 3 UV system (Merck, St. Louis, MO, USA). Acetonitrile and formic acid were purchased from Merck (Merck, St. Louis, MO, USA) and phytohormonal standards and isotopic labelled internal standards were provided by Olchemim Ltd. (Olomouc, Czech Republic).

### Experimental design and sample processing

For this study, experimental substrates were prepared by mixing clean soil and standard α-HCH, β-HCH and δ-HCH isomers to achieve a 50 mg/kg dry weight mixture of each, with a control soil sample prepared in the same way but without HCH. Next, two-year-old *A. glutinosa* saplings were planted in triplicate for all experimental and control variants and then placed outdoors in an unshaded site with instant access to water. The saplings purchased at the regular forestry nursery (Lesoškolky, Vestecká 999, 250 01 Brandýs nad Labem-Stará Boleslav, Czech Republic). This nursery is a licensed source of plant reproductive material. No further permissions are needed under Czech law to purchase saplings. The nursery declared the purchased saplings were *A.glutinosa*. We confirm that the experimental work performed with the plant material in this study complies with relevant institutional, national, and international guidelines and legislation.

After three months, 10.0 ± 0.5 g of soil was sampled from each pot for analysis of α-HCH, β-HCH and δ-HCH isomers, with an equal amount of soil used for dry mass determination (see below). The *A. glutinosa* saplings were then removed from the pots and divided into four sections, i.e. root, trunk, branches and leaves. Each part of the sapling was then weighed, after which it was milled in liquid nitrogen and the resultant fine powder used for HCH analysis, dry weight determination (both using 5.0 ± 0.5 g of biomass) and phytohormonal analysis (see below).

Samples for HCH extraction were agitated in a horizontal shaker at 200 rpm for 24 h in 10 ml of a 1:1 (v/v) acetone:hexane mixture for soil, and 5 mL of 1:1 (v/v) acetone:hexane for sapling biomass. The supernatant was then collected and anhydrous sodium sulphate added, after which the mixture was vortexed. One ml of extract was then taken and enriched with 10 μl of internal deuterated γ-HCH standard to cover ionisation instability prior to analysis (see below). Samples for dry mass determination were dried for three hours at 105 °C and then reweighed.

Rhizosphere soil, obtained by shaking the roots, and any remaining soil stuck to the roots was placed in a 50-ml test tube containing 35 ml of sterile phosphate buffer and then shaken for 5 min. The resultant solution was then centrifuged for 10 min at 4000×*g* at 4 °C to obtain biomass for DNA extraction (see below).

### Chemical analysis

Prior to mixing the experimental substrates, the purity of the standard isomer solutions was assessed using a RSH/Trace 1310/TSQ8000 Gas chromatography-tandem mass spectrometry (GC-MS/MS) assembly with a DB-5 ms column (PAL, Switzerland; ThermoFisher Scientific, USA). The same assembly was also used to assess the concentrations of HCH and ClB isomers in the various experimental samples. In each case, the limit of quantification (LOQ) was set at < 0.01 µg/g (dw) for all analytes. The standard deviation is up to 20% due to the complexity of the biological matrix.

Phytohormone analysis was performed using an Acquity® I-Class ultra-high-performance liquid chromatograph (UHPLC; Waters, Milford CT, USA) coupled with a Xevo TQ-XS MS/MS assembly (Waters, UK), using the isotope dilution method described by Šimura et al.^54^. Values under the limit of detection (LOD) were replaced by the values representing 95% of the detection limit for analysis.

### DNA extraction and real-time quantitative PCR

DNA extraction of soil and rhizosphere samples was undertaken in duplicate using the DNeasy power Soil KIT (Qiagen, Netherlands). DNA yield and quality were assessed using a Qubit fluorometer (Thermo Fisher Scientific, USA) and agarose gel electrophoresis.

The *lin* genes were assessed using the primers LinA-F (5′AGCTCAACGGATGCATGAACT3′), LinA-R (5′ GGCGGTGCGAAATGAATG3′), LinB-F (5′ACCACGGGCCGAATGC3′), LinB-R (5′ACCGTGATTTCGGTCTGGTTT3′), LinB-RT-F (5′GCGATCCGATCCTCTTCCA3′), LinB-RT-R (5′GCATGATATTGCGCCACAGA3′), LinD-F (5′GAACTGTTCCACTTCGTGTTCTCA3′), and LinD-R (5′GGTCACGCCCTTCTCCATTA3′)^50,55,56^. Total bacterial biomass was assessed using the *16S rDNA* gene and the primers U16SRT-F (5′ACTCCTACGGGAGGCAGCAGT3′) and U16SRT-R (5′TATTACCGCGGCTGCTGGC3′)^57^. All quantitative PCR (qPCR) reactions were run on a LightCycler 480 Real-Time PCR System (Roche, Switzerland), using white 96-well plates to increase sensitivity. The PCR program used was that described in our previous study^14^. The relative abundance of genes indicating total bacterial biomass (*16S rDNA*), dehydrochlorinase (*linA*), haloalkane dehalogenase (*linB*, *linB*-*RT*) and reductive dechlorinase (*linD*) were then presented as a heatmap table^58^.

### Amplicon 16S rRNA sequencing

The V4 region of the bacterial *16S rDNA* gene was amplified using the primers 530F (5′TGCCAGCMGCNGCGG3′) and 802R (5′TACNVGGGTATCTAATCC3′), while for fungal abundance, the *ITS2* region was amplified using the primers ITS3-F (5′ GCATCGATGAAGAACGCAGC3′) and ITS4-R (5′ TCCTCCGCTTATTGATATGC3′) at a final volume of 50 µL^59,60^. The amplicons were cleaned using the Agencourt Ampure XP system (Beckman Coulter, USA) and the Ion Torrent platform (Thermo Fisher Scientific, USA) was used for sequencing analysis. Barcoded sequencing adapters were ligated to the PCR products using the Ion Xpress Plus gDNA fragment library kit with Ion Xpress barcode adapters (Thermo Fisher Scientific, USA) and the samples analysed using the Ion PGM Hi-Q Sequencing Kit using an Ion 314 Chip (Thermo Fisher Scientific, USA).

The raw Ion Torrent reads were first processed using QIIME 2 v.2021.8 software^61^, after which the raw sequence data were demultiplexed and quality filtered using the q2‐demux plugin followed by denoising with DADA2^62^, with reads below 250 bp being removed. Taxonomy was assigned to each amplicon sequence variant (ASV) using the q2‐feature‐classifier^63^ classify-sklearn naive Bayes taxonomy classifier against the Silva 138 database^64^, after which Mitochondria and Chloroplasts were removed. Accuracy of classification was evaluated against an artificial MOCK community sample. The QIIME 2 outputs were then processed using the phyloseq R package^65^.

### Statistical analysis

The effect of each HCH isomer on sapling growth parameters was evaluated using one-way ANOVA with post-hoc Tukey tests, using the Origin software package v.2019b (OriginLab Corporation, USA). Prior to analysis, all data were subjected to Levene’s test to assess homogeneity of variance. All statistical analyses were performed at a significance level of α > 0.05. Data for phytohormonal analysis were first log-transformed and then subjected to principal components analysis (PCA) to describe the general data structure. The non-parametric Mann–Whitney test was then used to calculate significant differences in phytohormonal content between the control and HCH-polluted saplings.

## Results

### HCH treatment

#### Soil remediation

**Figure 1:**
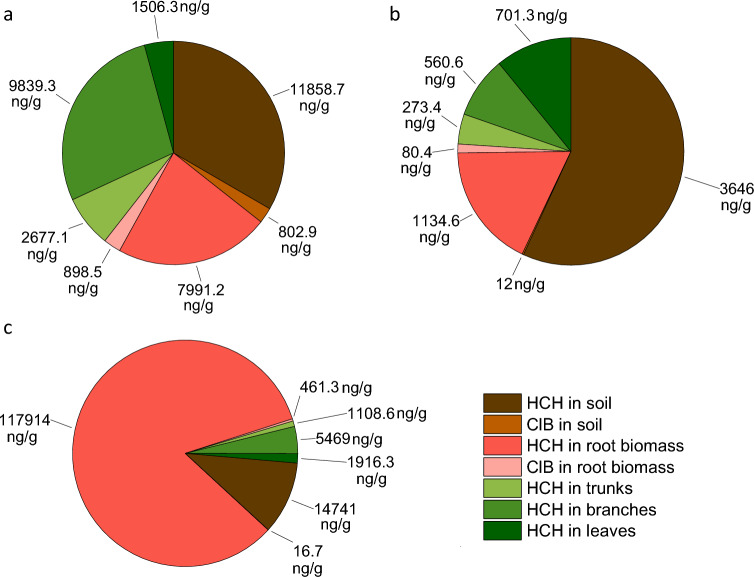
Concentrations of HCH isomers and metabolites (dry weight) in leaves, branches, roots and trunks of *A. glutinosa*; (**a**) α-HCH; (**b**) β-HCH; (**c**) δ-HCH.

HCH removal efficiency varied depending on the isomer, with degradation of each isomer differing with its persistence and physiological properties (e.g. solubility, volatility). Highest removal efficiency was observed in the group treated with β-HCH (90.26%), followed by α-HCH (64.85%) and δ-HCH (57.08%) (Fig. 1).

Highest ClB metabolite levels in soil were registered for α-HCH, with 10 metabolites (1,2-; 1,3-; 1,4-diClB, 1,2,3 -;1,2,4-triClB 1,2,4,5-; 1,2,3,5-; 1,2,3,4-tetraClB, pentaClB; hexaClB) found at a total concentration of 802.9 ng/g (Fig. 1). In comparison, lowest ClB metabolite levels were recorded in β-HCH polluted soils, with a total concentration of 12 ng/g dominated by hexaClB. Soils with δ-HCH contained 16.7 ng/g of ClB metabolites, mainly comprising the pentaClB and hexaClB isomers.

#### Sapling root biomass

The root biomass of saplings treated with α-HCH contained 7991.2 ng/g of the isomer, and a total concentration of 898.5 ng/g ClB, both metabolites matching those found in the soil. A similar decrease in metabolite concentrations was also observed for the β-HCH isomers, with root concentrations reaching 1134.63 ng/g and total metabolite levels just 80.43 ng/g (primarily hexaClB, pentaClB and 1,2,4-triClB).

#### Above-ground sapling parts

In general, concentrations of HCH were low in trunks, branches and leaves. Nevertheless, saplings exposed to α-HCH had concentrations reaching 2677.16 ng/g in trunks, 9839.33 ng/g in branches and 1506.30 ng/g in leaves, indicating that, once taken up by the roots, the isomer is subsequently transported throughout the sapling (Fig. 1). Interestingly, highest concentrations were determined in branches, where, presumably, no further processing of the original substance took place as no metabolites were detected. Subsequently, the isomer is transported to the leaves where further metabolism or volatilisation does occur. It should be noted, however, that there is a possibility that hitherto unknown metabolites are formed in all sapling parts.

Overall, less HCH and metabolites were recorded in sapling parts treated with the β-HCH isomer, with levels reaching just 273.36 ng/g in trunks, 560.60 ng/g in branches and 701.30 ng/g in leaves. Likewise, total metabolite concentrations were negligible, with just 1,2- and 1,4-diClB and 1,2,4-triClB detected in the trunks and hexaClB in the branches.

Results for the δ-HCH isomer were like those for the α-HCH isomer, with trunks containing 1108.63 ng/g, branches 5469 ng/g and leaves 1916.30 ng/g. Once again, there were higher levels in the branches than in other aboveground parts, though metabolites were only detected in trunks and not in branches or leaves.

##### Phytohormones

**Figure 2:**
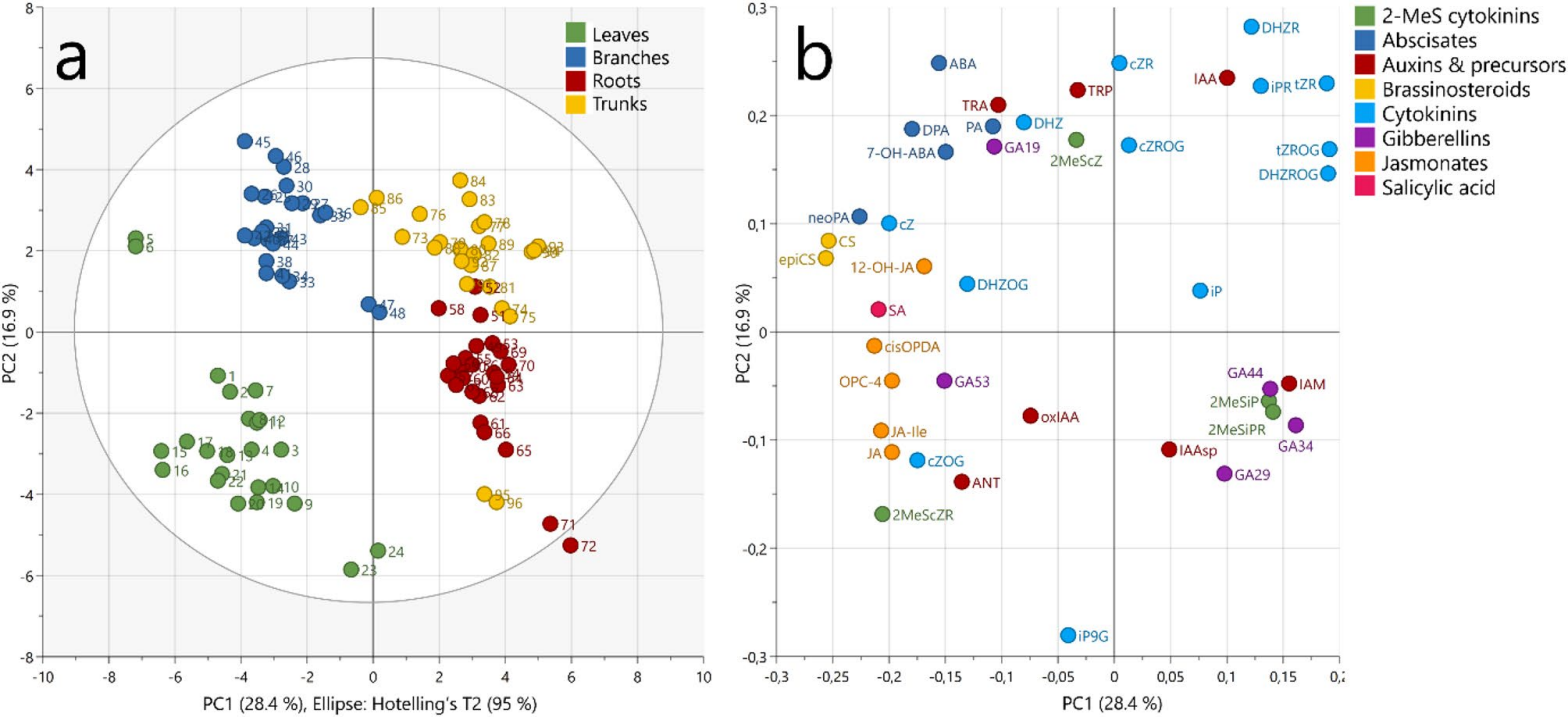
Variability of *A. glutinosa* saplings in plant hormone concentrations calculated by principal component analysis (PCA). In the score plot (**a**), each data point represents an individual plant sample coordinated by the principal components PC1 and PC2, both expressing total variability 45.3%. The similar samples, each coloured by a plant organ, are clustered close to each other. The numbers nearby the data points express the sample identifiers, whose meanings are explained in Table S1. The ellipse expresses the 95% limit of multivariate t-distribution (Hotelling’s T-squared distribution). The similarities of the samples are given by the concentrations of plant hormones depicted in the loading plot (**b**). The plant hormones co-localising with the samples on the same side of the scatter plot are abundant in these samples. Conversely, the plant hormones on the opposite side of the scatter plot are low in their concentration. The plant hormones are coloured by the corresponding phytohormonal group.

**Figure 3:**
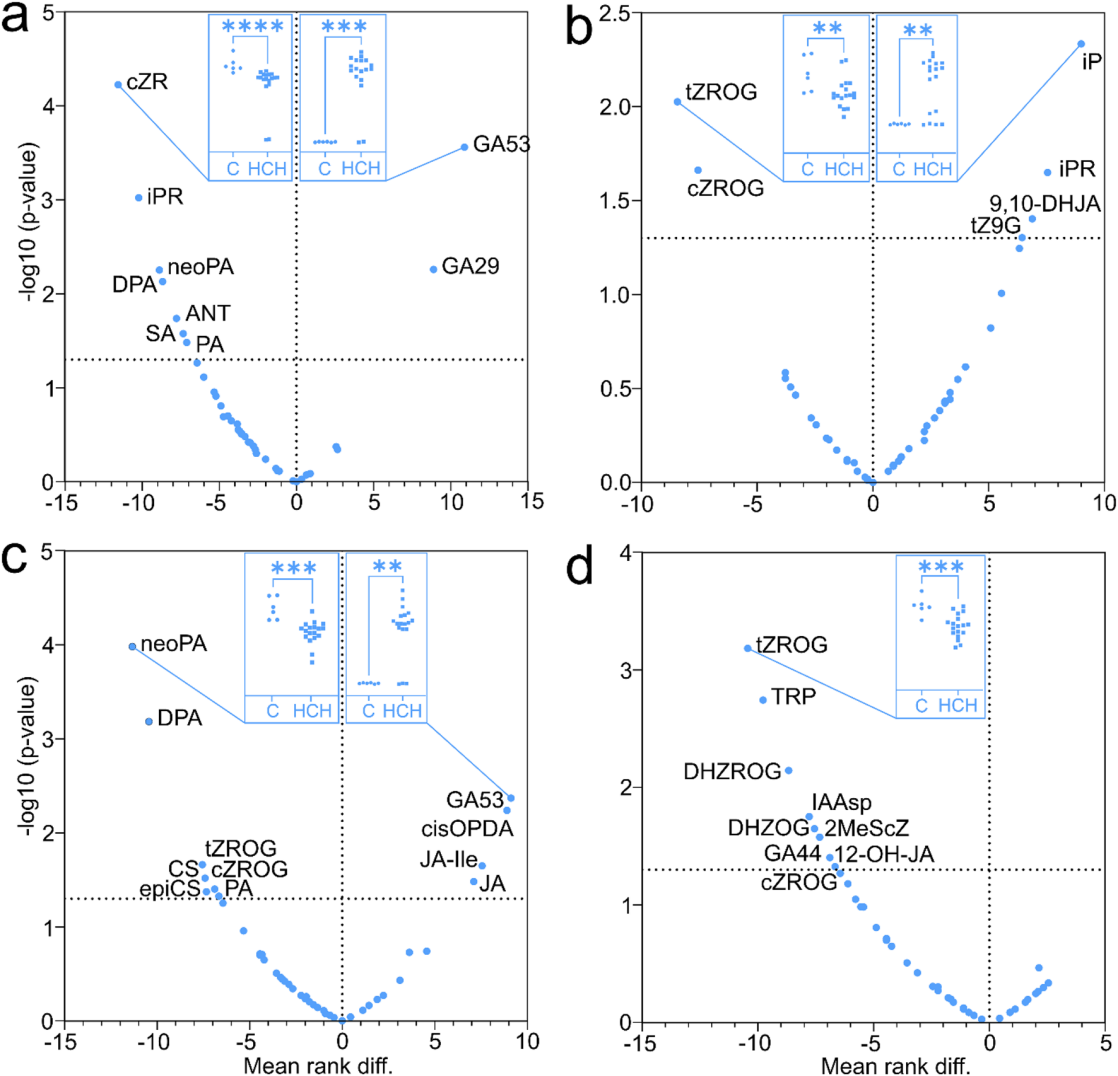
Effect of HCH on phytohormone concentration levels in the different parts of *A. glutinosa*; (**a**) branches, (**b**) trunk, (**c**) leaves and (**d**) roots. The blue circles in each volcano plot represent the plant hormones distributed according to the mean rank difference of the concentration between control (C) and HCH-treated groups (horizontal axis) and the negative logarithm of p-value calculated by Mann–Whitney test (vertical axis). The horizontal dashed line delimits the significance level of α = 0.05. The blue boxes inside each volcano plot shows the data distribution of the most significantly different plant hormone concentrations (with the highest absolute value of both mean rank difference and negative logarithm of p-value) in each individual sapling. The asterisks indicate the statistical significance at level of α = 0.05 (p-value 0 ‘****’0.0001 ‘***’0.001, ‘**’0.01).

Overall, 72 plant hormones and metabolites in eight classes were identified in tissues growing in contaminated and control soils (Fig. 2). The roots, for example, were characterised by highest concentrations of 2-methylthio CK and indole-3-acetamid. In comparison, highest concentrations of IAA (bioactive AUX) and the majority of CK were detected in sapling trunks. Branches and leaves typically had high concentrations of BR, accompanied by increased levels of ABA, JA and SA. CK in all sapling organs was downregulated in trees growing on polluted soil, whatever the isomer (Fig. 3). The saplings also contained metabolites of GA, with a significant increase in the precursor GA53 and the catabolite GA29 in stressed trees, along with upregulated JA and downregulated abscisates (Fig. 3a).

### Bacteria

Soil and rhizosphere samples produced 530 common bacterial taxa. Of these, 93 taxa were found in the rhizosphere but not in soil, and 126 in soil samples only (Fig. 1 supplementary). Soil bacterial community structure was similar in all samples treated with HCH.

**Figure 4:**
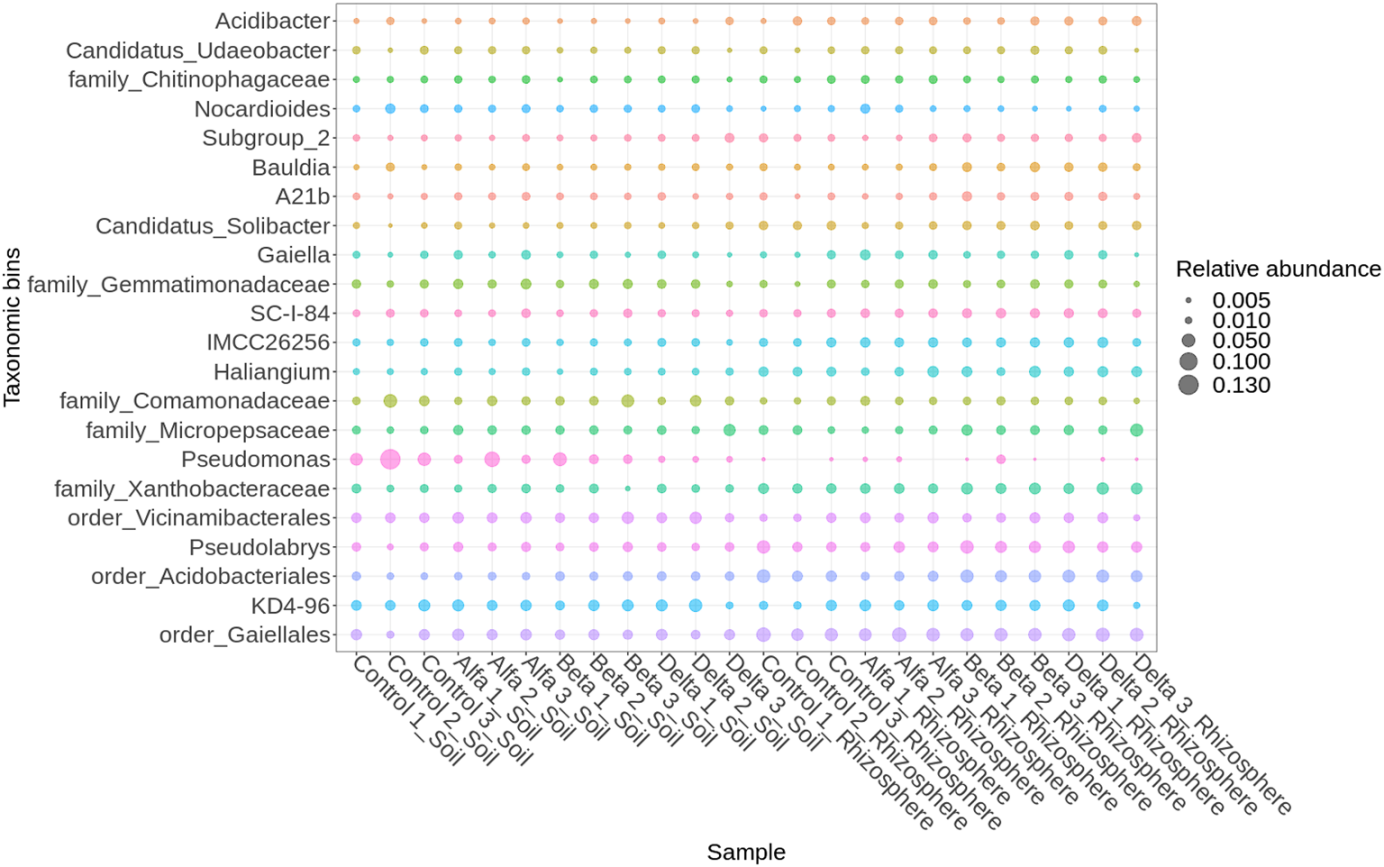
Relative abundance (mean > 0.01) of bacteria in soil and rhizosphere samples.

The dominant bacterial taxa in soil samples were, in order, *Pseudomonas*, the *unclassified Chloroflexi group KD4-96, Comamonadaceae, Nocardioides* and *Gemmatimonadaceae*. While *Pseudomonas* was dominant, with abundance levels highest in control samples, levels decreased noticeably in samples treated with HCH (Fig. 4).

Bacterial abundance was similar in all rhizosphere population samples, though with some exceptions (Fig. 4). For example, rhizosphere *Pseudomonas* levels were significantly lower than those in soil for all HCH treatments, with lowest abundance for the δ-HCH isomer, despite being detected at highest levels in the root biomass (Fig. 1). In contrast, *Pseudolabrys*, *Gaiellales (Gaiella)*, *Acidobacteriales, Haliangium* and *Bauldia* were the dominant contributors to biomass in the rhizosphere.

### Fungi

**Figure 5:**
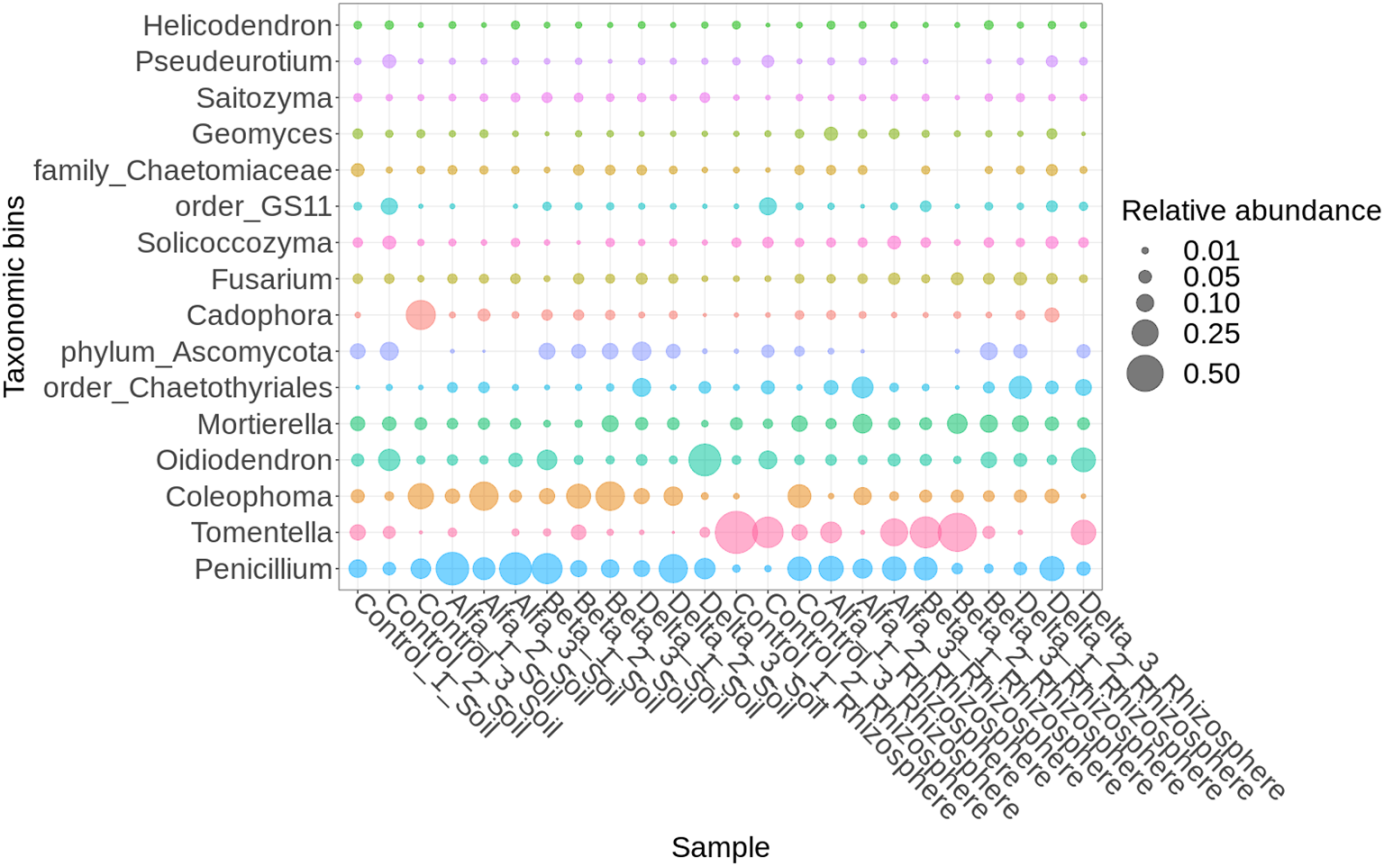
Relative abundance (mean > 0.01) of fungi in soil and rhizosphere samples.

Soil and rhizosphere samples contained 138 common fungal taxa, with 33 taxa found in the rhizosphere but not in soil and 86 found in soil only (Fig. 1 supplementary). The abundance of *Penicillium* and *Coleophoma* was higher in the soil than in the rhizosphere, while levels of *Mortierella* were higher in the rhizosphere (Fig. 5). While *Tomentella* was generally dominant in rhizosphere samples, samples treated with δ-HCH had the lowest concentrations and β-HCH samples and the control the highest.

### Functional genes involved in HCH biodegradation

The *lin* genes were found in all samples, with higher relative quantities in soil than the rhizosphere (Table 1). Interestingly, the *linA* gene was not detected in most rhizosphere samples but was present at low quantities in all soil samples. The *linB* gene was detected at high levels in soil samples, especially in samples treated with β-HCH. Overall, highest levels of the *linB-RT* gene were found in soil samples treated with the β-HCH isomer, while very little was recorded in control samples and none at all in soil treated with α-HCH, suggesting that the β-HCH isomer biodegrades in soil^44^. Finally, *linD* genes were detected at intermediate relative quantities in all rhizosphere samples, but were rarely detected in soil samples.

**Table 1:**
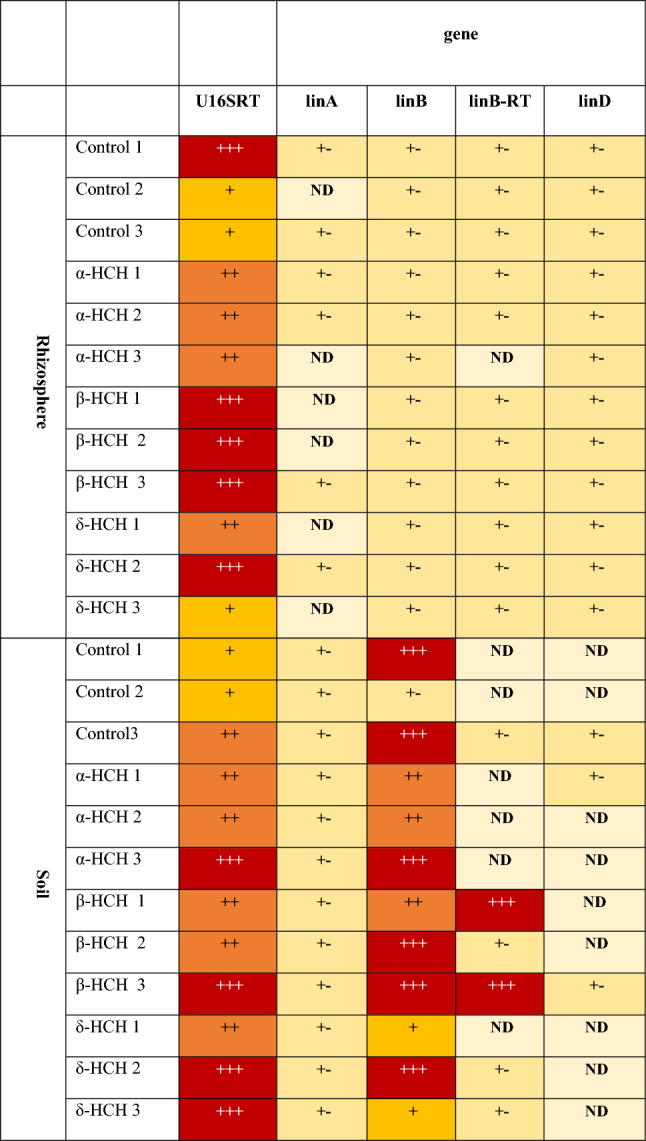
Relative abundance of genes indicating total bacterial biomass (*16S rDNA*), dehydrochlorinase (*linA*), haloalkane dehalogenase (*linB, linB-RT*) and reductive dechlorinase (*linD*) in rhizosphere and soil samples (average of duplicate samples).

The colour scale indicates the relative quantity of a given marker: red (+ + +) highest, orange (+ +) high, yellow ( +) intermediate, (+ -) low and *ND* not detected or below the LOQ.

## Discussion

This study provided several important findings regarding the impact of HCH pollution on *A. glutinosa* saplings and its associated soil microbiota, with implications for understanding the effects of persistent organic pollutants (POPs) on plant growth, microbial communities,phytohormone regulation, and applicability of HCH concentration in tree biomass for elucidation of HCH subsurface contamination (HCH phytoscreening).

### HCH removal efficiency

HCH removal efficiency varied with type of HCH isomer, with β-HCH exhibiting highest removal efficiency (90.26%), followed by α-HCH (64.85%) and δ-HCH (57.08%). Surprisingly, a high amount of isomer β-HCH was removed in this experiment, contrary to its persistent properties. In contrast, the removal of 64.58% of α-HCH is consistent with the findings of the study by Liu et al.^66^, where 47.3% was removed under hydroponic conditions in the presence of wheat. Chen et al.^67^ investigated the removal of β-HCH at realistic concentration levels in different plant species in constructed wetlands, including *Acorus calamus, Canna indica, Thalia dealbata,* and *Pontederia cordata*, and found decontamination efficiency against β-HCH in water ranging from 90.86 to 98.17%. Kidd et al.^40^ designed a greenhouse experiment to evaluate HCH dissipation and microbial parameters among rhizosphere and bulk soil of two contrasting plants, *Cytisus striatus* and *Holcus lanatus*, demonstrating the potential of these plant species in the degradative removal of α-HCH from the soil. The Wetland + system, comprising plant species such as *Phragmites* sp*., Phalaris* sp*., Typha* spp*., Sparganium erectum, Juncus* spp*., Glyceria fluitans*, among others, demonstrated high efficacy in HCH removal^68^. Specifically, it attained impressive removal rates of 96.8% for ClB and 81.7% for total HCH over a 12-month period. The low removal efficiencies of δ-HCH compared to the study by Košková et al.^51^ may be due to young age of the A. glutinosa seedling in their experiment. The differences in presented results can be attributed to differences in the persistence and physical and chemical properties of each HCH isomer, such as solubility and volatility. As the δ-HCH group is the most lipophilic (log K_ow_ = 4.14), soil composition may have negatively affected the availability of this isomer group to saplings and microbiota^69^. Simultaneously, this significant decline may be caused by the environmental influences. These findings were demonstrated in a study by Liu et al.^70^, where, when comparing the initial β-HCH concentration with the final concentration in the unplanted variant, this concentration was reduced by 50%. It is important to acknowledge, however, that the seedlings in this study were exposed to freshly dissolved HCH isomers, while at actual contaminated sites, these isomers may undergo chemical transformation due to interactions with environmental factors such as soil, water and air.

### Metabolite levels in HCH polluted soils

Lowest ClB metabolite levels were recorded in β-HCH polluted soils. It is important to note that transportation of HCH does not always follow the ‘water diffusion pattern’ through the environment as its octanol–water partition coefficient is close to 4 (log K_ow_)^69^. In contrast, soils containing δ-HCH had 16.7 ng/g of ClB metabolites, primarily consisting of the pentaClB and hexaClB isomers. In comparison, α-HCH is more susceptible to metabolic transformation in soils, most likely through bacterial activity in cooperation with plants. Indeed, numerous studies have reported high degradation rates of α-HCH through a range of microorganisms^71–73^.

Based on the available literature, it is evident that ClB can be metabolized by microorganisms and possibly by plants. Trapp et al.^74^ demonstrated the presence of polar metabolites of ClB in plants, indicating their potential metabolism by plants. Furthermore, Middeldorp et al.^75^ and Kurt & Spain^76^ provided evidence of ClB biotransformation under specific conditions, suggesting microbial metabolism. The studies by Wang et al.^77^ and Langenhoff et al.^78^ also indicated the biodegradation potential of ClB by microbial consortia under different conditions. Moreover, Ramanand et al.^79^ and Nishino et al.^80^ provided insights into the reductive dehalogenation and degradation of ClB by bacteria, further supporting the microbial metabolism of these compounds.

### HCH uptake and transport in saplings

The highest concentration of HCH isomers was recorded in the root part of plants. This is attributed to the proximity of contaminated soil and to the lipophilicity of the individual isomers and the root structures. The lipophilic compounds are able to enter the root tissue, where in most cases they remain bound to the lipophilic layer of the root epidermis^81^. The uptake of HCH by plants has been recorded under laboratory^70^ and field conditions^16^. The root biomass of saplings treated with α-HCH contained 7991.2 ng/g α-HCH and a total ClB concentration of 898.50 ng/g, mirroring soil metabolite levels, and exhibited a rapid decrease in metabolite concentrations, possibly due to factors such as low uptake, rapid degradation, transportation or transformation within the roots. A similar decline in metabolite concentrations was also evident for the β-HCH isomers. In terms of above-ground sapling components, HCH concentrations were generally low in trunks, branches, and leaves. Saplings exposed to α-HCH, however, exhibited elevated concentrations in leaves, suggesting that once absorbed by the roots, the isomer is subsequently transported throughout the sapling. Intriguingly, substantial concentrations were identified in branches, where it is presumed that minimal further processing of the original substance occurs as no metabolites were detected. Conversely, the isomer is then transported to the leaves, where additional metabolism or volatilization may take place. This transport is supported by the vapor pressure of the isomer, which may explain the transport from the root to the aerial parts of plants^82^. It is essential to acknowledge the possibility of the formation of yet undiscovered metabolites within all sapling parts. In contrast, sapling parts treated with the β-HCH isomer displayed lower HCH and metabolite levels, while the results for δ-HCH closely resembled those observed for α-HCH. Similarly, higher concentrations were identified in the branches compared to other aboveground parts, though ClB metabolites were only detected in the trunks and not in branches or leaves.

### Phytohormonal responses

The greatest difference in phytohormone concentration levels was observed between the various sapling organs, most likely due to differences in organ function and developmental patterns over time and space. For example, the roots showed notable levels of 2-methylthio CK and indole-3-acetamid, which serve as indicators of bacterial activity in the rhizosphere^83,84^. The highest concentrations of IAA (bioactive AUX), and the majority of CK, were detected in sapling trunks, presumably as homeostasis between trunk AUX and CK regulates various physiological functions, including apical dominance^85^. The phytohormonal profile of branches and leaves displayed enrichment of BR, typically associated with the meristems of actively growing young organs^86^. Additionally, elevated levels of ABA, JA and SA in the branches and leaves indicated a potential response to abiotic stress conditions^87^. Across all sapling organs, CK levels were generally downregulated in trees cultivated in contaminated soil, irrespective of the HCH isomer applied. This downregulation may be linked to early senescence as a reaction to stress induced by phytoremediation efforts^88^. Notably, isopentenyladenine and isopentenyladenosine in the sapling trunks showed exceptions to this trend, likely associated with CK transport mechanisms^89,90^. Furthermore, our study identified metabolites of GA in the *A. glutinosa* samples, which have previously been described as components of the 13-hydroxy biosynthetic GA pathway found in buds of mountain alder *Alnus incana* subsp. *tenuifolia*^91,92^. In this study, stressed trees exhibited a significant increase in precursor GA53 and the catabolite GA29, alongside upregulated JA and downregulated ABA. The notable concentration of JA suggests that signalling pathways involved in abiotic stress responses had been activated. It is noteworthy that components of the JA signalling pathway have previously been reported as interacting antagonistically with ABA in *Arabidopsis* (rockcress, Brassicaceae), which could explain the observed lower concentration of ABA metabolites in our study on *A. glutinosa*^93,94^. ABA is also known to act antagonistically with GA, which could account for the increase in GA levels observed in our stressed trees^95^.

### Microbial communities in soil and rhizosphere

Bacterial community analysis revealed a comprehensive array of 530 common bacterial taxa in both soil and rhizosphere samples. Furthermore, our findings revealed the prevalence of *Nocardioides, Chloroflexi* and *Gemmatimonadaceae*, which have also been reported as abundant taxa in soils contaminated with organochlorine pesticides (OCPs)^96^. Interestingly, the unclassified *Chloroflexi group KD4-96* has frequently been detected in soils contaminated with heavy metals^97^, with abundance correlating strongly with iron (Fe) and aluminium (Al) concentrations^98^. In contrast, *Pseudolabrys, Gaiellales (Gaiella), Acidobacteriales, Haliangium* and *Bauldia* were all dominant contributors to biomass in the rhizosphere, with *Pseudolabrys*, order *Rhizobiales*, being nitrogen-fixing bacteria that establish a symbiotic relationship with plant roots, similar to *Bauldia*. Additionally, *Gaiella* plays a crucial role as an organic matter decomposer and actively participates in carbon cycling^99^. Of the fungi, *Tomentella* was generally dominant in rhizosphere samples, while soil samples treated with δ-HCH had very low concentrations and soils with β-HCH and the control group having highest levels. Tedersoo et al. identified 40 species of putatively ectomycorrhizal fungi from seven sites dominated by *A. glutinosa*, with the ectomycorrhizal fungi *Tomentella* aff*. Sublilacina* most prevalent under saline stress conditions^100^. *Tomentella* contain melanins, which may act as a boundary between fungal cells and their environment, protecting them against physical, chemical and/or biological stressors^101^. Our findings revealed that five taxonomic bins: *Ferribacterium, Methylotenera, Fluvicola, env.OPS_17* and *Exiguobacterium* exhibited statistically significant differences exclusively within soil samples between experimental treatments and control counterparts (Fig. 7 supplementary and Table S2). Conversely, in other sample types, the observed distinctions did not attain statistical significance.

### Functional genes related to HCH biodegradation

Several functional genes related to HCH biodegradation were detected, including *linA*, *linB* and *linD*. The *lin* genes were found in all samples, with higher quantities in soil than in the rhizosphere. Presence of the *linB* gene was most pronounced in soil samples, particularly in those exposed to β-HCH. Lal et al. (2010) also reported *linB* occurring at higher levels than *linA* in most soil samples from a highly contaminated HCH dump site^44^. The presence of *linB* genes in soils with β-HCH may indicate the potential for efficient biodegradation of HCH isomers, aligning with the observed relationship between *linB* genes and β-HCH removal efficiency. Sharma et al. (2006) demonstrated that Haloalkane Dehalogenase LinB is responsible for β- and δ-HCH transformation in *Sphingobium indicum *B90A, highlighting the enzymatic basis for the degradation of HCH isomers, including β-HCH. The key function of *linB* is the breakdown of the most resistant HCH isomer, β-HCH^102^. Furthermore, the similarity of *linB* to other enzymes (as opposed to *linA*, which is unique) may result in the selection of more *linB* genes from unidentified bacteria present in the samples^44^. These findings suggest that soil microbial communities possess the genetic potential for HCH biodegradation, with *linB* a prominent gene involved in β-HCH degradation. The presence of these genes in the rhizosphere indicates the potential for plant–microbe interactions in HCH remediation.

### Study limitations

We recognise that the absence of an unplanted control group in this study may be a limitation. While we endeavoured to investigate the bioaccumulation potential of HCH isomers in *A. glutinosa* saplings and their associated microbial communities comprehensively, the inclusion of an unplanted control group would have provided valuable insights into the background levels of HCH decontamination via exterior physical conditions. Despite this limitation, our study offers significant insights into HCH isomer bioaccumulation patterns in relation to *A. glutinosa* saplings and their associated responses, contributing to the broader understanding of HCH persistence in the environment and its ecological implications.

### Discussion of hypotheses

Our first hypothesis, which suggested that the δ-HCH isomer would show lowest bioaccumulation in *A. glutinosa* saplings, followed by α-HCH and β-HCH, was not supported by the findings. The δ-HCH isomer did show, in contrast, highest bioaccumulation potential, followed by α-HCH and β-HCH. These differences in bioaccumulation can be attributed to variations in chemical properties and affinities of individual HCH isomers for plant uptake.

Our second hypothesis proposed that highest HCH concentrations would be found in the root system of *A. glutinosa* saplings, with decreasing concentrations in the trunk, branches and leaves, reflecting the plant’s uptake and translocation mechanisms. This hypothesis was largely confirmed by our results, which showed that HCH concentrations were indeed highest in the roots, with sequentially decreasing levels in above-ground plant parts. This distribution pattern aligns with the plant’s mechanisms for HCH uptake and translocation.

Our final hypothesis suggested that the introduction of HCH isomers would lead to changes in the composition and abundance of microbial communities, with specific bacterial species responding differently to different HCH isomers, potentially indicating their ability to degrade or interact with the contaminants. The observed microbial community dynamics in response to HCH isomers were either subtle or not statistically significant, suggesting that specific populations may not have exhibited pronounced variations in their response to different HCH isomers. This outcome implies that factors beyond HCH isomer type, such as soil conditions or other environmental variables, may have played a more influential role in shaping microbial communities during the study. Further research including a more comprehensive exploration of these factors will be necessary to improve our understanding of the intricate interactions between microbial communities and HCH isomers in contaminated environments.

## Conclusion

*A.glutinosa* saplings showed clear uptake of all HCH isomers, with highest quantities detected in the roots and lowest in the leaves. Owing to its physicochemical properties, the δ-HCH isomer was the most persistent in soils and the most strongly bound to *A. glutinosa* roots, with α-HCH the second major isomer recorded in soils treated with β-HCH and δ-HCH. All metabolites were present at highest concentrations in the α-HCH isomer treatment, suggesting that α-HCH may be subject to degradation over the longer term. All HCH isomers were found at highest proportions in the soil, with relatively little found in root biomass, suggesting that degradation of HCH isomers by soil bacteria occurs mainly through the upstream pathway. However, high levels of the *linD* gene in rhizosphere bacteria, also confirmed HCH degradation via downstream pathways. Overall, there was no significant difference in the abundances of bacterial and fungal consortia between treated and control samples. Similarly, there were no significant differences between soil and rhizosphere microorganisms. Phytohormone analysis indicated that *A. glutinosa* reacts to HCH contamination through changes in the stress hormones CK, JA, abscisate and GA.

Our findings have broad practical implications for a range of fields. Firstly, they offer valuable insights for environmental remediation efforts, aiding in the development of more effective and sustainable phytoremediation strategies for HCH-contaminated soils. Secondly, this research contributes to the assessment of soil and ecosystem health in areas affected by POPs, thereby informing ecosystem restoration and conservation strategies. Moreover, these insights could drive advancements in bioremediation technologies, offering nature-based solutions for contaminated land restoration. While our results provide preliminary information on the phytoremediation potential of *A. glutinosa* trees, further studies will be needed at actual contaminated sites to fully understand their ability to remediate contaminated soils.

### Supplementary Information

Supplementary Information.

## Data Availability

The data presented in this study are available under conditions on request from the corresponding authors Pavel Hrabák and Alena Sevcu. The sequencing data is available through the following link http://www.ncbi.nlm.nih.gov/bioproject/937905.
