# Supplementary material for: Assessing HCH isomer uptake in Alnus glutinosa: implications for phytoremediation and microbial response
Source: Sci Rep. 2024 Feb 20;14:4187. doi: 10.1038/s41598-024-54235-1 (PMC10879209; doi:10.1038/s41598-024-54235-1)
Supplement: Supplementary file 1 — Supplementary Information. [file 41598_2024_54235_MOESM1_ESM.docx]

*
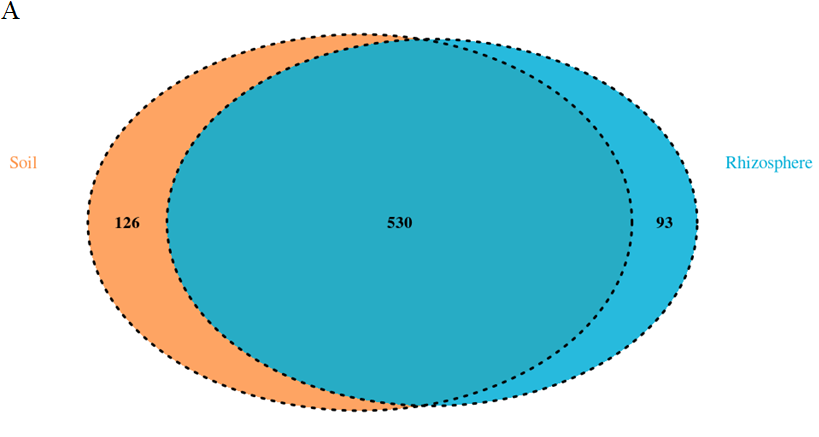

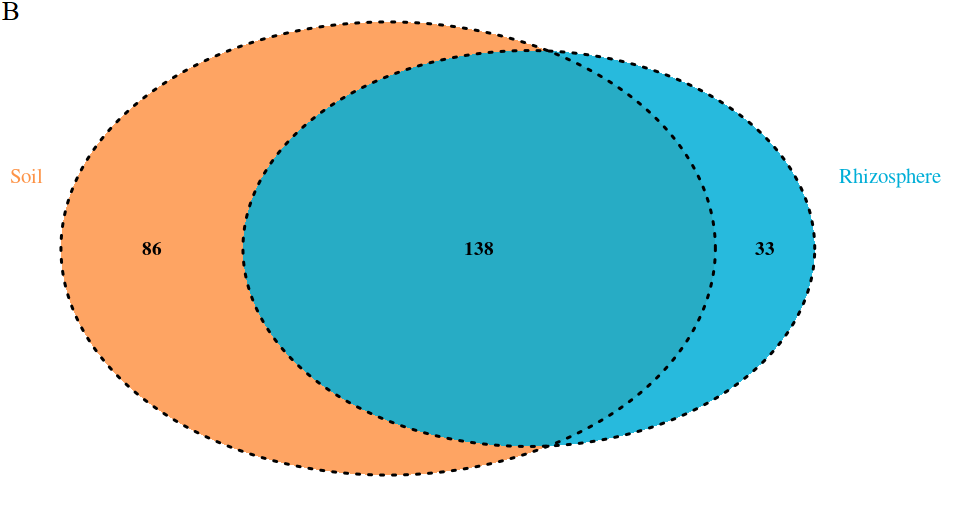
*

Figure 1 supplementary. Venn diagram showing common microbial (A) and fungal (B) populations shared by the soil and rhizosphere samples.


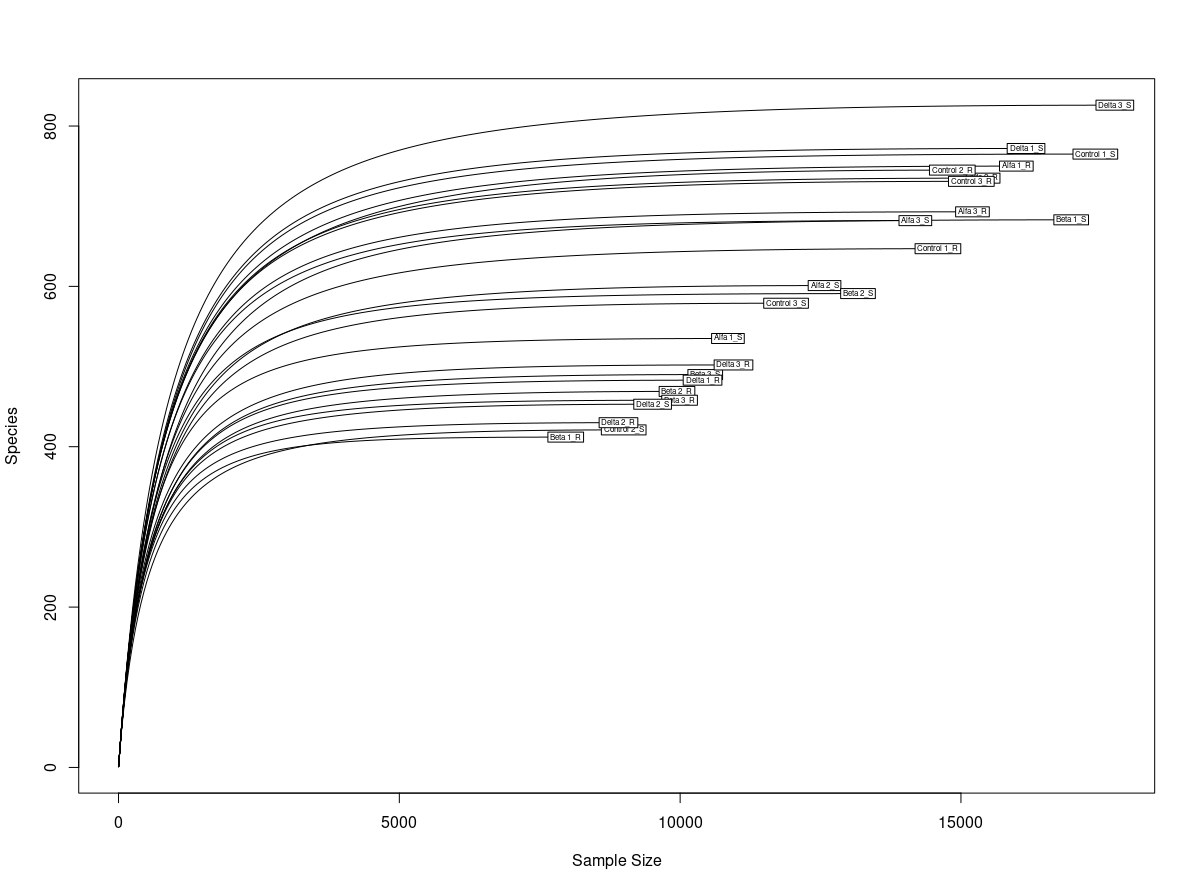


Figure 2 supplementary. Rarefaction curves for 16S rRNA gene amplicons
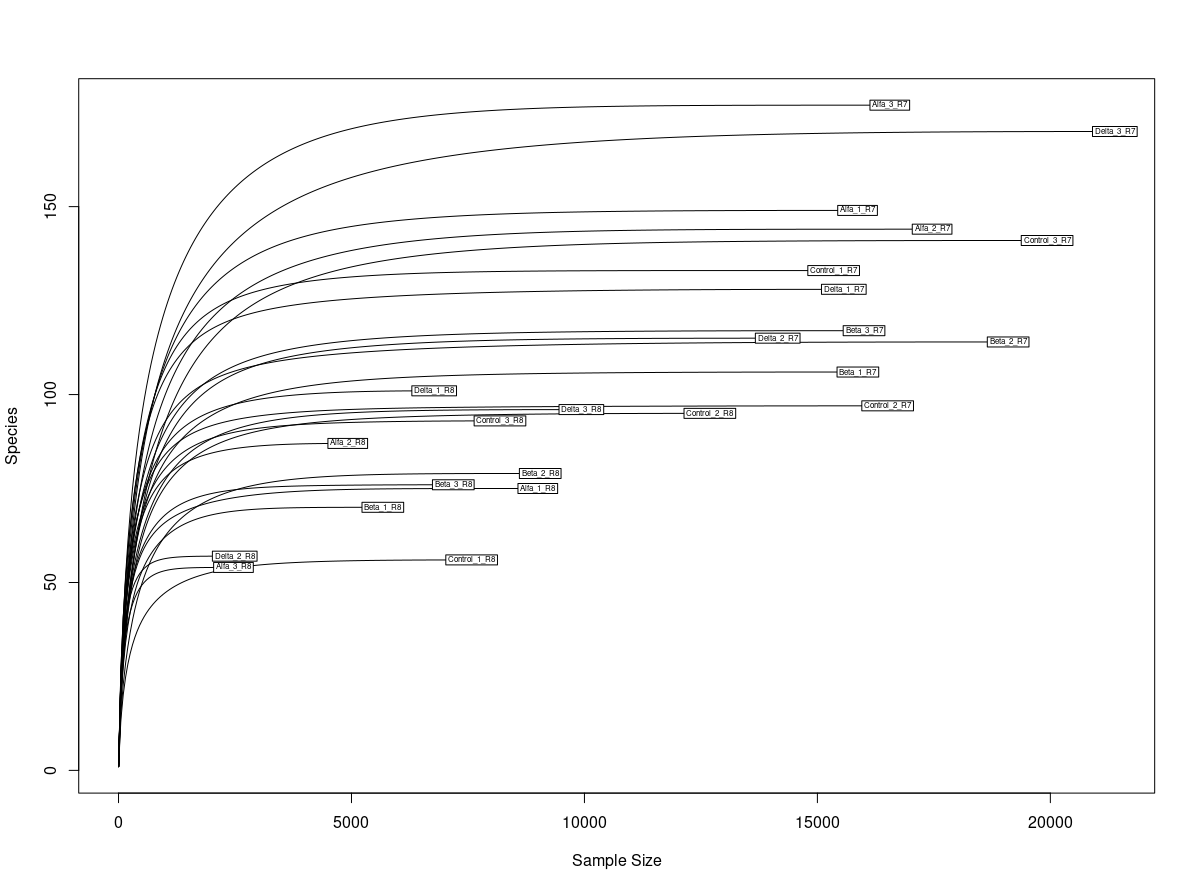


Figure 3 supplementary. Rarefaction curves for ITS gene amplicons


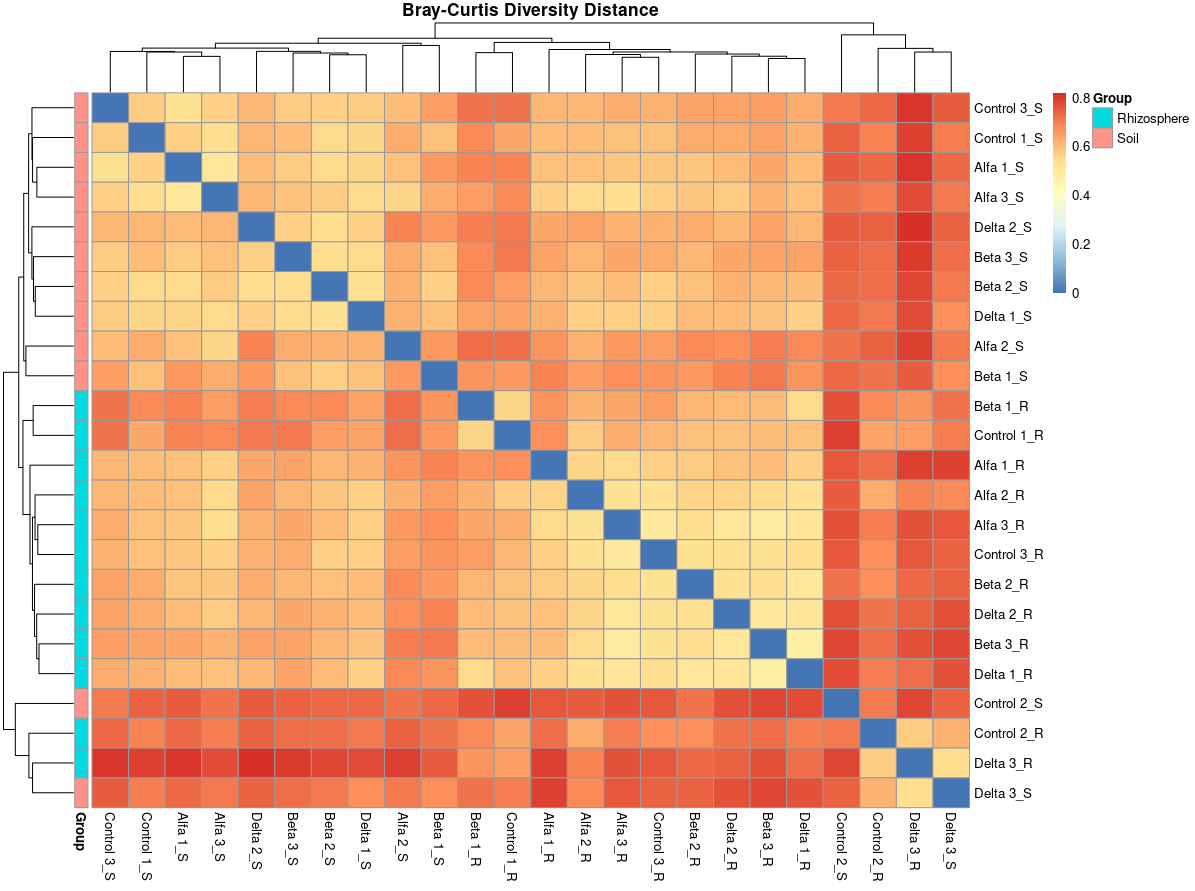


Figure 4 supplementary. Bray-Curtis diversity distance based on 16S rRNA gene sequencing data.


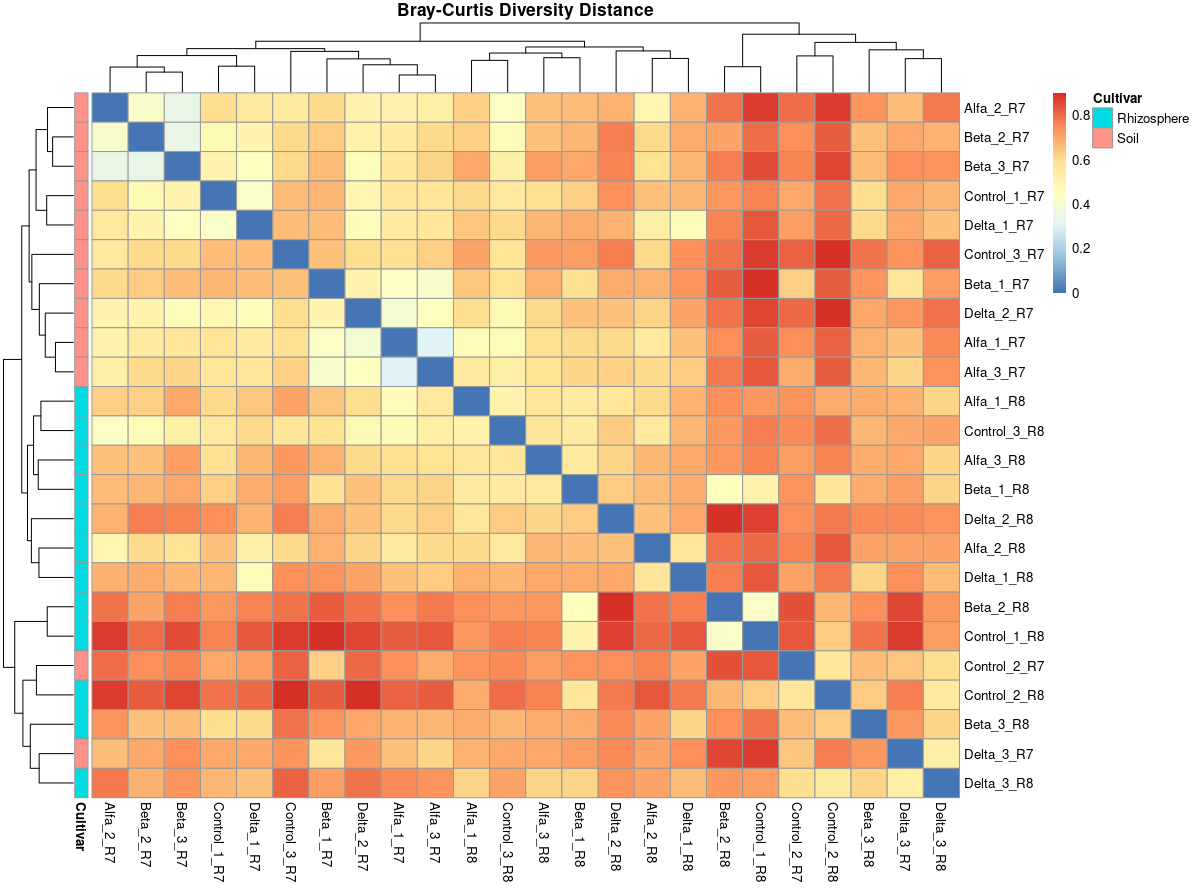


Figure 5 supplementary. Bray-Curtis diversity distance based on ITS gene sequencing data.

Figure 6 supplementary**.** Effect of HCH-pollution on *A. glutinosa* growth parameters; (A) sapling height; (B) root biomass; (C) trunk biomass; (D) branch biomass; (E) leaf biomass.


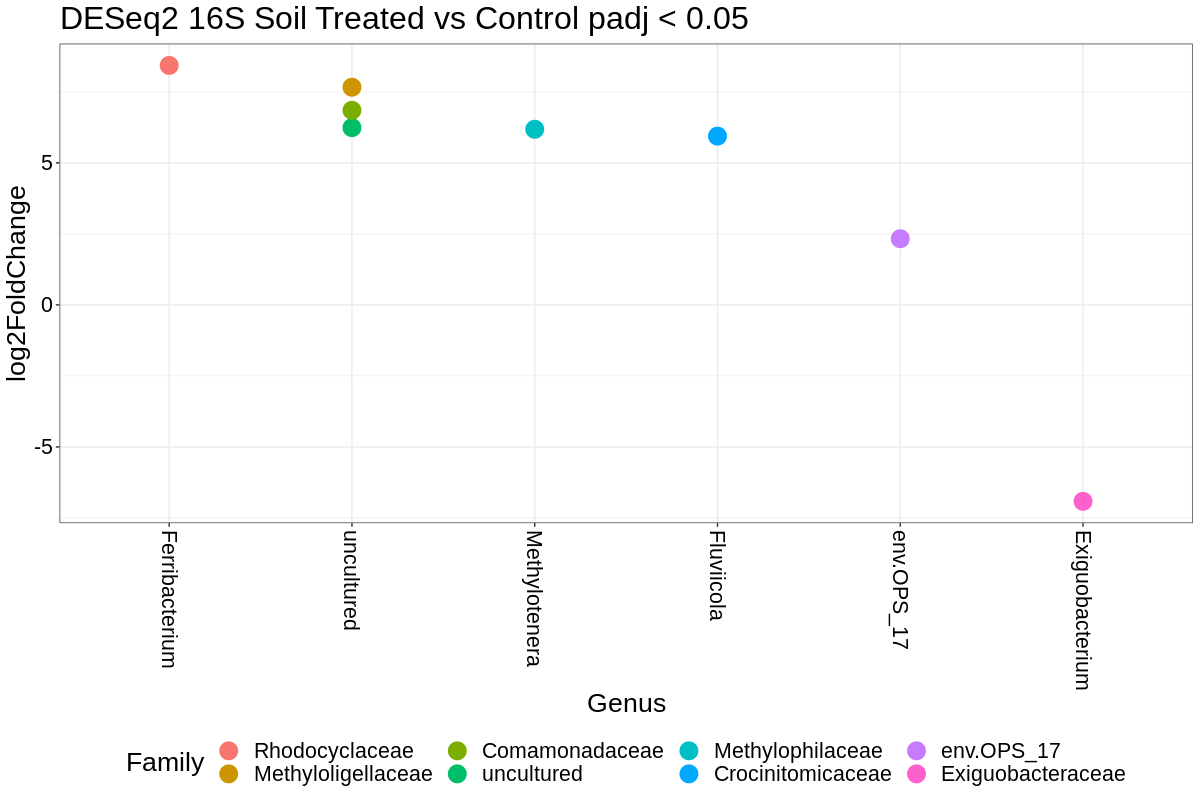


Figure 7 supplementary**.** DEseq2 16s soil treated samples vs Control

Table S1. Explanatory variables of A. glutinosa samples in plant hormone analysis

| # Sample | Organ | Treatment |
| --- | --- | --- |
| 1 | Leaves | control |
| 2 | Leaves | control |
| 3 | Leaves | control |
| 4 | Leaves | control |
| 5 | Leaves | control |
| 6 | Leaves | control |
| 7 | Leaves | α-HCH |
| 8 | Leaves | α-HCH |
| 9 | Leaves | α-HCH |
| 10 | Leaves | α-HCH |
| 11 | Leaves | α-HCH |
| 12 | Leaves | α-HCH |
| 13 | Leaves | β-HCH |
| 14 | Leaves | β-HCH |
| 15 | Leaves | β-HCH |
| 16 | Leaves | β-HCH |
| 17 | Leaves | β-HCH |
| 18 | Leaves | β-HCH |
| 19 | Leaves | δ-HCH |
| 20 | Leaves | δ-HCH |
| 21 | Leaves | δ-HCH |
| 22 | Leaves | δ-HCH |
| 23 | Leaves | δ-HCH |
| 24 | Leaves | δ-HCH |
| 25 | Branches | control |
| 26 | Branches | control |
| 27 | Branches | control |
| 28 | Branches | control |
| 29 | Branches | control |
| 30 | Branches | control |
| 31 | Branches | α-HCH |
| 32 | Branches | α-HCH |
| 33 | Branches | α-HCH |
| 34 | Branches | α-HCH |
| 35 | Branches | α-HCH |
| 36 | Branches | α-HCH |
| 37 | Branches | β-HCH |
| 38 | Branches | β-HCH |
| 39 | Branches | β-HCH |
| 40 | Branches | β-HCH |
| 41 | Branches | β-HCH |
| 42 | Branches | β-HCH |
| 43 | Branches | δ-HCH |
| 44 | Branches | δ-HCH |
| 45 | Branches | δ-HCH |
| 46 | Branches | δ-HCH |
| 47 | Branches | δ-HCH |
| 48 | Branches | δ-HCH |
| 49 | Roots | control |
| 50 | Roots | control |
| 51 | Roots | control |
| 52 | Roots | control |
| 53 | Roots | control |
| 54 | Roots | control |
| 55 | Roots | α-HCH |
| 56 | Roots | α-HCH |
| 57 | Roots | α-HCH |
| 58 | Roots | α-HCH |
| 59 | Roots | α-HCH |
| 60 | Roots | α-HCH |
| 61 | Roots | β-HCH |
| 62 | Roots | β-HCH |
| 63 | Roots | β-HCH |
| 64 | Roots | β-HCH |
| 65 | Roots | β-HCH |
| 66 | Roots | β-HCH |
| 67 | Roots | δ-HCH |
| 68 | Roots | δ-HCH |
| 69 | Roots | δ-HCH |
| 70 | Roots | δ-HCH |
| 71 | Roots | δ-HCH |
| 72 | Roots | δ-HCH |
| 73 | Trunks | control |
| 74 | Trunks | control |
| 75 | Trunks | control |
| 76 | Trunks | control |
| 77 | Trunks | control |
| 78 | Trunks | control |
| 79 | Trunks | α-HCH |
| 80 | Trunks | α-HCH |
| 81 | Trunks | α-HCH |
| 82 | Trunks | α-HCH |
| 83 | Trunks | α-HCH |
| 84 | Trunks | α-HCH |
| 85 | Trunks | β-HCH |
| 86 | Trunks | β-HCH |
| 87 | Trunks | β-HCH |
| 88 | Trunks | β-HCH |
| 89 | Trunks | β-HCH |
| 90 | Trunks | β-HCH |
| 91 | Trunks | δ-HCH |
| 92 | Trunks | δ-HCH |
| 93 | Trunks | δ-HCH |
| 94 | Trunks | δ-HCH |
| 95 | Trunks | δ-HCH |
| 96 | Trunks | δ-HCH |

Table S2. DESeq2 16S Soil Treated vs Control

| Base  Mean | log2  FoldChange | lfcSE | stat | pvalue | padj | Kingdom | Phylum | Class | Order | Family | Genus |
| --- | --- | --- | --- | --- | --- | --- | --- | --- | --- | --- | --- |
| 9.971 | 6.239 | 1.596 | 3.908 | 0.00009 | 0.012 | d__Bacteria | Proteobacteria | Alphaproteobacteria | Rhodospirillales | uncultured | uncultured |
| 9.632 | 6.183 | 1.713 | 3.609 | 0.00031 | 0.024 | d__Bacteria | Proteobacteria | Gammaproteobacteria | Burkholderiales | Methylophilaceae | Methylotenera |
| 45.820 | 8.431 | 1.620 | 5.206 | 0.00000 | 0.000 | d__Bacteria | Proteobacteria | Gammaproteobacteria | Burkholderiales | Rhodocyclaceae | Ferribacterium |
| 5.624 | -6.916 | 1.826 | -3.787 | 0.00015 | 0.014 | d__Bacteria | Firmicutes | Bacilli | Exiguobacterales | Exiguobacteraceae | Exiguobacterium |
| 31.571 | 2.329 | 0.515 | 4.525 | 0.00001 | 0.002 | d__Bacteria | Bacteroidota | Bacteroidia | Sphingobacteriales | env.OPS_17 | env.OPS_17 |
| 15.329 | 6.851 | 1.714 | 3.997 | 0.00006 | 0.010 | d__Bacteria | Proteobacteria | Gammaproteobacteria | Burkholderiales | Comamonadaceae | uncultured |
| 26.898 | 7.663 | 1.749 | 4.381 | 0.00001 | 0.002 | d__Bacteria | Proteobacteria | Alphaproteobacteria | Rhizobiales | Methyloligellaceae | uncultured |
| 8.217 | 5.942 | 1.568 | 3.790 | 0.00015 | 0.014 | d__Bacteria | Bacteroidota | Bacteroidia | Flavobacteriales | Crocinitomicaceae | Fluviicola |
